# Cannabis Use and Resting State Functional Connectivity in the Aging Brain

**DOI:** 10.3389/fnagi.2022.804890

**Published:** 2022-02-10

**Authors:** Karli K. Watson, Angela D. Bryan, Rachel E. Thayer, Jarrod M. Ellingson, Carillon J. Skrzynski, Kent E. Hutchison

**Affiliations:** ^1^Institute of Cognitive Science, University of Colorado Boulder, Boulder, CO, United States; ^2^Department of Psychology and Neuroscience, University of Colorado Boulder, Boulder, CO, United States; ^3^Department of Psychology, University of Colorado Colorado Springs, Colorado Springs, CO, United States; ^4^Department of Psychiatry, University of Colorado Anschutz Medical Campus, Aurora, CO, United States

**Keywords:** cannabis, marijuana, default network, fMRI, elderly, cognition, cognitive, Alzheimer’s

## Abstract

Several lines of evidence suggest that older adults (aged 65+) sharply increased their cannabis use over the last decade, highlighting a need to understand the effects of cannabis in this age group. Pre-clinical models suggest that cannabinoids affect the brain and cognition in an age-dependent fashion, having generally beneficial effects on older animals and deleterious effects on younger ones. However, there is little research on how cannabis affects the brains of older adults or how older adults differ from younger adults who use cannabis. Resting state functional connectivity (rsFC) measures provide sensitive metrics of age-related cognitive decline. Here we compared rsFC in older adults who are either regular users of cannabis or non-users. We found stronger connectivity between sources in the hippocampus and parahippocampal cortex, and targets in the anterior lobes of the cerebellum in older adult cannabis users relative to non-users. A similar pattern of strengthened connectivity between hippocampal and cerebellar structures was also present in 25–35 year old non-users in comparison to 60–88 year old non-users. These findings suggest that future studies should examine both the potential risks of cannabinoids, as well as a potential benefits, on cognition and brain health for older adults.

## Introduction

The last decade has seen unprecedented shifts in cannabis availability, products, and patterns of use. Many legal markets offer cannabis products with ever-increasing variety and potencies. For example, the Δ^9^-tetrahydrocannabinol (THC) potency of confiscated cannabis flower tripled from 1995 to 2012 (4–12% THC), and legal markets now sell cannabis flower that often exceeds 20% THC (ElSohly et al., 2016). It is unknown how widely available forms of cannabis are used by, or affect, individuals differentially across the lifespan. Several lines of evidence suggest that older adults (aged 65+) sharply increased their cannabis use during this period, highlighting a need to understand the effects of cannabis among this age group. The proportion of adults 65 and older in the United States reporting cannabis use quadrupled between 2007 and 2015 (Han et al., 2017; Han and Palamar, 2018) and increased another 75% between 2015 and 2018 (Han and Palamar, 2020). Despite these changes, there is little research on how cannabis may affect older adults, or how older adults who use cannabis differ from younger adults who use cannabis (Hutchison et al., 2019; Weinstein and Sznitman, 2020).

In pre-clinical models, low doses of THC appear to be neuroprotective and even reverse age-related cognitive decline in older animals, while at the same time, these same THC doses appear to have deleterious effects in younger animals (Bilkei-Gorzo et al., 2017). Specifically, THC administration to aged mice increased hippocampal spine density and synaptic connectivity and enhanced cognitive performance, while THC administration in younger animals worsened cognitive performance and had no effect on spine density. Other studies investigating the effects of agonists to the CB1 receptor, the same receptor stimulated by THC, have found that CB1 receptor agonist administration to old rats improves spatial memory, reduces age-related inflammation, and induces hippocampal neurogenesis (Marchalant et al., 2008, 2009). The seemingly opposite effects of cannabis constituents on older versus younger animals may be mediated by age-related changes in the endocannabinoid system that include an age-related decrease in CB1 receptor binding and gene expression in the cerebral cortex, limbic structures, and hippocampus (Berrendero et al., 1998). In summary, pre-clinical studies suggest that there are age-related changes in the endocannabinoid system of the brain, and that cannabinoids may benefit cognition in older animals (Piyanova et al., 2015).

Though most human studies of the effect of cannabis on the brain involve adolescents and emerging adults, there are a few studies in middle-aged or older adults that generally suggest little or no effect of cannabis use on cognitive function (Weinstein and Sznitman, 2020). Two studies examining the effects of recreational cannabis on cognition in mid-life adults found no evidence of cognitive decline in cannabis users (Dregan and Gulliford, 2012; McKetin et al., 2016). Another found that current marijuana use in mid-life was associated with decreases in verbal recall and processing speed measures, but not executive function (Auer et al., 2016). On the other hand, two longitudinal studies that compared pre- and post-exposure performance reported that cannabis was associated with improved cognitive task performance in middle-aged adults (∼50 years old) and these improvements were accompanied by changes in brain activity (Gruber et al., 2016, 2018). With respect to adults over the age of 60, one study found that cannabis use was not associated with differences in cognition or brain structure (Thayer et al., 2019). Clearly, the literature is limited but the evidence thus far suggests either no association between cannabis use and cognition, brain structure, or function or perhaps a small positive effect in older adults.

Resting state functional connectivity (rsFC), that is, the simultaneous activity of brain regions, is a measure that is sensitive to aging and cognitive decline (Ferreira and Busatto, 2013; Varangis et al., 2019; Zonneveld et al., 2019). As such, rsFC may provide insight regarding the effects of cannabis use in the aging population. Here, we examine the effects of self-reported cannabis use on rsFC in older adults. First we investigate differences between cannabis users and non-users using orbitofrontal cortex as the seed region, given that both anatomical (Filbey et al., 2014; Chye et al., 2017; Lorenzetti et al., 2019) and functional (Filbey et al., 2014) studies have found associations between this region and cannabis use in middle aged adults (mean age ∼30 years). Second, we investigate networks involving the posterior cingulate, given its central role in the default mode network (Greicius et al., 2003) and fMRI evidence that it is modulated by cannabis use (Bossong et al., 2013; Pujol et al., 2014). Finally, based on animal and human evidence implicating hippocampal changes with cannabis use (Yücel et al., 2008; Ashtari et al., 2011; Demirakca et al., 2011; Bilkei-Gorzo et al., 2017) and aging (Miller and O’Callaghan, 2005; Barrientos et al., 2015; Leal and Yassa, 2015), we test whether networks involving this region differs between older cannabis users and non-users. To facilitate the interpretation of our findings in older adults, we additionally investigate rsFC differences found in older vs. younger adult non-users. Based on the neuroprotective effects of cannabinoids found in animal studies described above, it was hypothesized that the cannabis-using group would show altered rsFC in hippocampal networks compared to non-users, and that these alterations would be consistent with those found in younger versus older participants.

## Materials and Methods

### Experimental Procedures

The behavioral and neuroimaging data were collected as part of two different studies. The data were combined to increase the sample size for the current study. The first dataset (*n* = 186) was collected as part of a study aimed at characterizing the effect of increasing exercise on psychological and cognitive function in older adults (1R01AG043452). Older adults 60 years of age were recruited and completed self-reported health and functioning measures, including items measuring cannabis use, objective cognitive functioning measures, and a functional magnetic resonance imaging (fMRI) session. A smaller group of younger adults (ages 25–35 years) was recruited to compare neurocognitive function, and completed the same baseline assessments as the older adults. The second dataset (*n* = 38) was collected as a pilot study to explore the effects of cannabis in older adults (R36DA040020). Older adults 60 years of age or older were recruited and screened as described previously (Thayer et al., 2019). Briefly, adults who reported consuming cannabis once per week for the last year or who reported never using cannabis completed a demographics questionnaire, basic questions about cannabis use, a cognition battery, and measures to identify alcohol use disorder, marijuana dependence, and depression.

### Participants

Participants were recruited through community advertisements, online resources such as Craigslist, ResearchMatch, outlets commonly frequented by older adults, and public records purchased from a marketing firm.

To determine eligibility for dataset 1, interested individuals were asked to complete a phone screen. Inclusion criteria were: (1) 25–35 years of age for younger adults or 60+ years of age for older adults; (2) not meeting physical activity recommendations, defined as reporting fewer than 80 min per week of moderate-to-vigorous intensity exercise over the past 6 months; (3) completion of the Pfeiffer Short Portable Mental Status Questionnaire (SPMSQ; Pfeiffer, 1975) with fewer than three errors; (4) willingness to be randomly assigned to condition (older adults only); (5) able to safely engage in moderate intensity exercise, as assessed by a study physician; (6) completion of a VO_2_ max test without evidence of cardiac or other abnormalities; and (7) intending to remain in the Boulder-Denver area for at least 6 months (older adults only). To determine eligibility for dataset 2 the following criteria were applied: (1) 60+ years of age, (2) <20 pack years of tobacco use; (3) absence of a history of alcohol or other substance use disorder other than cannabis use disorder.

For both datasets, individuals with uncontrolled diabetes (hemoglobin A1C > 7%), uncontrolled hypertension (systolic BP ≥ 160 mmHg and/or diastolic BP ≥ 100 mmHg), bipolar disorder, schizophrenia, dementia, Alzheimer’s disease, MRI contraindications, and/or body size exceeding MRI capacity were deemed ineligible. Individuals who were pregnant or taking antipsychotic medications during the screening process were also excluded. The Institutional Review Board of the University of Colorado Boulder approved all study procedures for both datasets (Protocol 13-0392 for dataset 1 and 15-0457 for dataset 2).

### Procedure

Written informed consent was obtained from all participants. Participants in dataset 1 completed a baseline health assessment, functional assessment, and medical and MRI screening. Participants in dataset 1 also completed an interview about their current fitness level, a test of physical function, and a physician-supervised treadmill familiarization activity with a 12-lead EKG. Participants in dataset 2 completed a single session of completing behavioral and cognitive measures and MRI.

#### Demographics

Participants self-reported their age, gender, race/ethnicity, and socioeconomic status at baseline.

#### Cannabis Use

Participants were asked a series of questions about cannabis use, including whether they currently use cannabis, and, if so, the frequency of use (e.g., “every day” to “less than monthly”).

### Magnetic Resonance Imaging Acquisition

Scan data were acquired on a Siemens 3T MRI scanner with a 32-channel head coil at the Intermountain Neuroimaging Consortium at the University of Colorado Boulder. Scan data for dataset 1 acquired before April 2016 were collected on a TRIO system. In April/May 2016 the TRIO system was upgraded to a Prisma Fit system; data acquired after May 2016 were collected on this system. Scanner type was included as a nuisance covariate in all fMRI analyses. Dataset 2 data were collected on the Prisma Fit system only.

For both datasets, field maps with reversed phase-encoded blips were acquired (*TR* = 7220 ms, *TE* = 73 ms, FOV = 248 mm × 248 mm). Each participant underwent a multi-echo MPRAGE (magnetization prepared rapid acquisition with gradient echo) T1 weighted anatomical scan (datasets 1 and 2: *TR* = 2530 ms, *TE* = 1.64 ms, flip angle = 7°, FOV = 256 mm × 256 mm). A resting state M-EPI scan was also acquired (dataset 1: *TR* = 460 ms, *TE* = 29 ms, multiband acceleration factor = 8, slices = 48; dataset 2: *TR* = 460 ms, *TE* = 27.2 ms, multibank acceleration factor = 8, slices = 56). During the resting state scan, participants were instructed to stare on a central fixation cross and relax for 8 min. Acquired images employed simultaneous image refocusing and multiband slice excitation (Feinberg and Setsompop, 2013). This newer method of spatial and temporal multiplexing has allowed for much faster sampling rates at <500 ms instead of ∼2 s, while still acquiring whole-brain coverage. This acquisition method also reduces high-frequency artifacts such as physiological noise, thereby increasing the signal-to-noise ratio by 60%.

### Data Preprocessing and Analysis

Susceptibility distortion correction was applied by the Mind Research Network using FSL topup to generate a susceptibility-induced off resonance field (Andersson et al., 2003; Smith et al., 2004). All subsequent MRI analysis and preprocessing was performed using the Matlab-based functional connectivity software CONN (Version 18.b) (Whitfield-Gabrieli and Nieto-Castanon, 2012; The MathWorks, 2018). After distortion correction, preprocessing steps included: realignment and unwarping, outlier identification (framewise displacement above 0.9 mm or global BOLD signal changes above 5 s.d.), head movement estimation, tissue segmentation (gray matter, white matter, CSF), normalization to MNI space, and smoothing with a Gaussian kernel of 8 mm full width half maximum (FWHM). We next applied CONN’s default denoising pipeline to the preprocessed acquisitions. We performed linear regression of potential confounds in the BOLD signal, implementing anatomical component-based noise correction procedures (aCompCor) (Behzadi et al., 2007). A band pass filter of 0.008–0.09 Hz and a linear detrending algorithm were applied after regression. For each subject, data were quality checked for gross artifacts or errors that may have been caused during preprocessing or denoising. Scans exhibiting excessive distortion or non-normal distributions of post-denoising connectivity values were discarded (*n* = 3, all older adult participants).

## Results

To examine the relationship of cannabis use to brain connectivity, we first examined rsFC values during resting state fMRI in our older participants (*n* = 221). Older adult participants were between 60 and 88 (mean 67.5 years, sd 5.65, 60.6% female). For this analysis, we contrasted brain activity between two main groups: Those who used cannabis once or more weekly (“users,” *n* = 43), and those who described themselves as “not current cannabis users” (“non-users,” *n* = 153). Participants who described themselves as “current cannabis users” but who also reported less than weekly cannabis use (*n* = 25) were excluded from the analysis. Handedness, race, and ethnicity were roughly balanced across participant groups (Supplementary Table 1). The older non-user group was female-dominated (66.5% female), whereas the older user group was not (39.5% female). Sex and scanner type were included as nuisance covariates in all models.

We began with seed-to-voxel analyses contrasting users and non-users in the older adult sample. Selecting *a priori* regions of interest based on previous literature, we tested three separate seeds of interest: Bilateral orbitofrontal cortex, bilateral posterior cingulate (given its association with the default network), and bilateral hippocampus. There were no significant differences in seed-to-voxel rsFC between older adult cannabis users and non-users when using orbitofrontal cortex or posterior cingulate as our seed regions.

**FIGURE 1:**
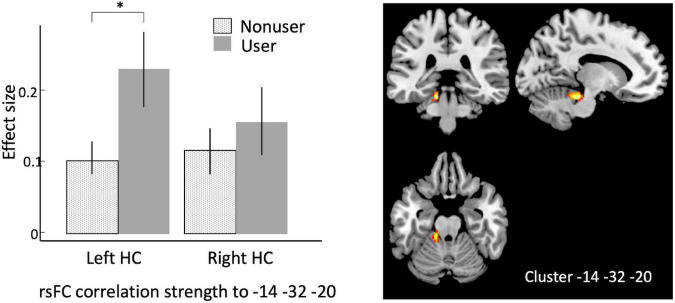
Seed-to-voxel analysis using bilateral hippocampi as seeds, contrasting older cannabis users vs. older cannabis non-users. Hippocampal seeds show stronger correlation to a cluster in left cerebellum at in older cannabis users vs. older non-users. Left, effect sizes of left vs. right hippocampus connectivity to the peak voxel in older non-users (stippled bar) and older users (solid bar); Right, warm colors represent significant voxels of heightened connectivity from hippocampus, overlaid on canonical anatomical scan (peak voxel at *x* = –14, *y* = –32, *z* = –20). HC, hippocampus. Asterisk indicates *p* < 0.00001, error bars indicate 95% C.l.

Using bilateral hippocampus as seed regions, a single cluster of voxels yielded rsFC values that were significantly associated with cannabis use. A cluster in left cerebellum (MNI coordinates –14, –32, –20; 101 voxels; *p*-FWE = 0.027) had significantly increased rsFC with left hippocampus in users vs. non-users (Figure 1 and Table 1).

**TABLE 1:** Composition of the single significant cluster in a seed-to-voxel analysis comparing aged users vs. aged non-users, using bilateral hippocampus as seeds.

Number of voxels	Region
58	Cerebellum lobules IV/V
24	Cerebellum lobule III
10	posterior parahippocampal gyrus, Left
9	Brainstem

**FIGURE 2:**
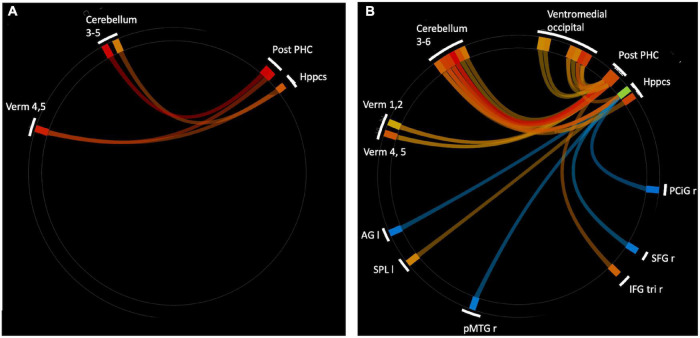
ROI-to-ROI analysis using hippocampal and parahippocampal cortex (anterior and posterior) seeds. **(A)** Significantly strengthened connections for aged users vs. aged non-users. There were no connections that were significantly stronger for aged non-users vs. users. **(B)** Significantly altered connections for younger (18–35 years) vs. older (60+ years) non-users. Warm colors indicate young > old, cool colors indicate old > young. Ventromedial occipital includes occipital fusiform, temporal occipital fusiform, and posterior temporal occipital fusiform. Abbreviations: r, right; I, left; Verm, vermis; Post-PHC, posterior parahioppocampal cortex; Hppcs, hippocampus; PCiG, paracingulate gyrus; SFG, superior frontal gyrus; IFG tri, interior frontal gyrus, pas triangularis; pMTG, middle temporal gyrus, posterior division; SPL, superior parietal lobule; AG, angular gyrus.

To expand on this finding, we next performed an analysis using the CONN built-in ROI atlas of 164 brain regions, based on the Harvard-Oxford atlas for cortical and subcortical regions, and the Automated Anatomical Labeling (AAL) atlas for cerebellar subregions. In this analysis, we chose six *a priori* seed regions consisting of bilateral hippocampus, posterior parahippocampal cortex (pPaHC), and anterior parahippocampal cortex (aPaHC). ROI-to-ROI analyses take the mean resting brain activity across all the voxels within each anatomically defined source ROI and correlates this with the mean brain activity across voxels in each anatomically defined target ROI. Consistent with our seed-to-voxel analysis, we found stronger connectivity with anterior cerebellum (lobules III and IV/V and vermis IV/V; Figure 2 and Table 2).

**TABLE 2:** Significantly altered connections comparing aged users vs. aged non-users in an ROI to ROI analysis, using bilateral anterior parahippocampus, bilateral posterior parahippcampus (pPaHC), and bilateral hippocampus as seed regions.

Source	Target	*T* value	*p*-FWE
Parahippocampal Gyrus, posterior division Left	Cerebellum 4 5 Left	4.79	0.0004
Parahippocampal Gyrus, posterior division Right	Vermis 45	3.93	0.016
Hippocampus Left	Vermis 45	3.52	0.049
Hippocampus Left	Cerebellum 3 Left	3.43	0.049

*All altered connections are users > non-users. Statistics shown are FWE corrected at the seed level. R, right; L, left; Cerebellum 45, Cerebellar lobules IV/V; Vermis 45, Vermis IV/V; Cerebellum 3, Cerebellar lobule III.*

In order to determine which pattern of connectivity might be more typical of the older adult brain, we next examined brain connectivity in these same regions comparing older adult non-users and the sample of younger adult non-users between the ages of 25 and 35 (*n* = 43, mean 28.9 years, sd 3.1). Our goal was to determine whether the brain connectivity differences observed in older users vs. non-users were consistent with a profile of accelerated aging, decelerated aging, or neither. We conducted an ROI-to-ROI analysis using bilateral hippocampus and anterior and posterior hippocampal cortex as the source regions and contrasted older non-users and younger non-users (*n* = 23, 52.2% female, Supplementary Table 1), using seed-based FDR-corrections as above. We found that, relative to older non-users, younger non-users had significantly stronger rsFC between pPaHC and anterior cerebellum (cerebellar lobules III and IV/V, vermis IV/V), similar to the stronger rsFC in older users vs. older non-users. Younger non-users also had significantly stronger rsFC between hippocampus and anterior cerebellum (cerebellar lobules III and IV/V, vermis IV/V). Thus, rsFC involving cerebellar lobules IV/V were particularly influenced by age, with strengthened rsFC to bilateral hippocampus, pPaHC, and aPaHC. There were also several altered connections between the source regions and ventromedial occipital cortex (Figure 2 and Table 3).

**TABLE 3:** Significantly altered connections comparing young adult non-users and aged non-users in an ROI to ROI analysis, using bilateral anterior parahippocampus, bilateral posterior parahippcampus, and bilateral hippocampus as seed regions.

Source	Target	*T*-value	*p*-FWE
Parahippocampal Gyrus, posterior division Right	Cerebellum 4 5 Right	5.11	0.0001
Parahippocampal Gyrus, posterior division Right	Cerebellum 4 5 Left	4.99	0.0001
Parahippocampal Gyrus, posterior division Right	Temporal Fusiform Cortex, posterior division Left	4.03	0.0036
Parahippocampal Gyrus, posterior division Right	Temporal Fusiform Cortex, posterior division Right	3.62	0.0127
Parahippocampal Gyrus, posterior division Right	Cerebellum 6 Right	3.5	0.0157
Parahippocampal Gyrus, posterior division Right	Vermis 4 5	3.21	0.0321
Parahippocampal Gyrus, posterior division Right	Cerebellum 6 Left	3.19	0.0321
Parahippocampal Gyrus, posterior division Right	Cerebellum 3 Right	3.05	0.0403
Parahippocampal Gyrus, posterior division Right	Temporal Occipital Fusiform Cortex Left	3.04	0.0403
Parahippocampal Gyrus, posterior division Right	Vermis 1 2	2.94	0.0495
Parahippocampal Gyrus, posterior division Left	Cerebellum 4 5 Left	5.8	0
Parahippocampal Gyrus, posterior division Left	Cerebellum 4 5 Right	5.16	0
Parahippocampal Gyrus, posterior division Left	Cerebellum 6 Left	4.72	0.0002
Parahippocampal Gyrus, posterior division Left	Temporal Fusiform Cortex, posterior division Left	4.2	0.0014
Parahippocampal Gyrus, posterior division Left	Cerebellum 6 Right	4.1	0.0017
Parahippocampal Gyrus, posterior division Left	Inferior Frontal Gyrus, pars triangularis Right	3.7	0.0064
Parahippocampal Gyrus, posterior division Left	Temporal Occipital Fusiform Cortex Left	3.58	0.0085
Parahippocampal Gyrus, posterior division Left	Vermis 1 2	3.53	0.0086
Parahippocampal Gyrus, posterior division Left	Cerebellum 3 Left	3.34	0.0152
Parahippocampal Gyrus, posterior division Left	Temporal Fusiform Cortex, posterior division Right	3.25	0.0185
Parahippocampal Gyrus, posterior division Left	Vermis 4 5	3.07	0.0279
Parahippocampal Gyrus, posterior division Left	Occipital Fusiform Gyrus Right	3.06	0.0279
Parahippocampal Gyrus, posterior division Left	Temporal Occipital Fusiform Cortex Right	2.97	0.0332
Parahippocampal Gyrus, posterior division Left	Occipital Fusiform Gyrus Left	2.96	0.0332
Parahippocampal Gyrus, anterior division Right	Cerebellum 4 5 Right	4.62	0.001
Hippocampus Right	Cerebellum 6 Left	3.53	0.0371
Hippocampus Right	Cerebellum 4 5 Right	3.51	0.0371
Hippocampus Left	Cerebellum 6 Left	4.27	0.0042
Hippocampus Left	Cerebellum 4 5 Left	4.08	0.0046
Hippocampus Left	Temporal Fusiform Cortex, posterior division Right	3.81	0.0079
Hippocampus Left	Cerebellum 6 Right	3.75	0.0079
Hippocampus Left	Cerebellum 4 5 Right	3.68	0.0083
Hippocampus Left	Paracingulate Gyrus Right	–3.35	0.0215
Hippocampus Left	Angular Gyrus Left	–3.23	0.0265
Hippocampus Left	Superior Parietal Lobule Left	3.2	0.0265
Hippocampus Left	Middle Temporal Gyrus, posterior division Right	–3.17	0.0265
Hippocampus Left	Superior Frontal Gyrus Right	–3.1	0.0301
Parahippocampal Gyrus, anterior division Left	Temporal Fusiform Cortex, posterior division Right	3.93	0.0162
Parahippocampal Gyrus, anterior division Left	Cerebellum 4 5 Left	3.53	0.035
Parahippocampal Gyrus, anterior division Left	Cerebellum 6 Right	3.33	0.0352
Parahippocampal Gyrus, anterior division Left	Temporal Fusiform Cortex, posterior division Left	3.33	0.0352
Parahippocampal Gyrus, anterior division Left	Cerebellum 4 5 Right	3.17	0.0479

*Positive t-statistics indicate young > old, negative indicate old > young. Statistics shown are FWE corrected at the seed level. Cerebellum 4 5, Cerebellar lobules IV/V; Ver45, Vermis IV/V; Cerebellum 3, Cerebellar lobule III; Cerebellum 6, Cerebellar lobule VI.*

**FIGURE 3:**
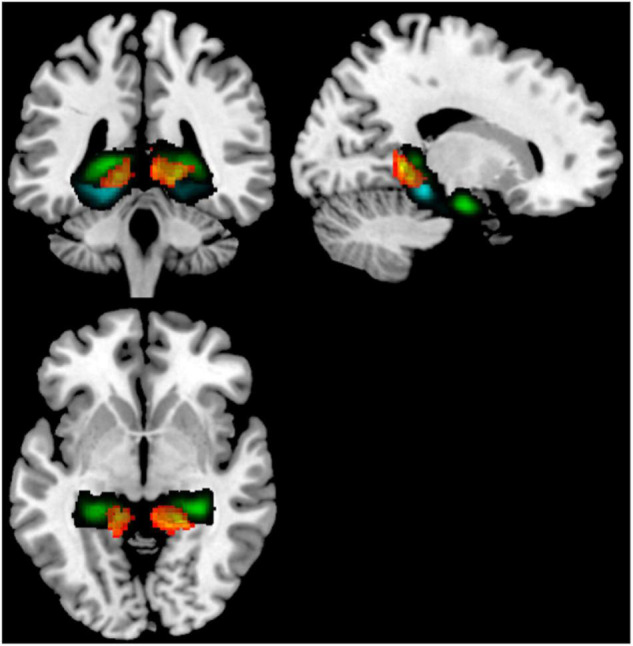
Seed-to-voxel analysis contrasting older users vs older non-users, illustrating increased rsFC from a cerebellum 4/5 seed to a cluster (warm colors) overlapping the hippocampus (green) and posterior parahippocampal cortex (cyan). Target regions derived from the Harvard-Oxford atlas. Illustrated slices from *X* = 184, *Y* = 135, *Z* = 132.

Because cerebellar lobules IV/V had such consistent altered rsFC across hippocampal and parahippocampal subregions in our comparison of younger non-users to older non-users, to determine specificity, we next performed a seed-to-voxel analysis contrasting older users to older non-users, using bilateral cerebellar lobules IV/V as seeds. This analysis revealed two fairly symmetric clusters of target voxels from these seeds, indicating stronger rsFC in younger vs. older non-users. These clusters were bilateral (*x* = –14, *y* = –50, *z* = 10; *x* = +12, *y* = –44, *z* = +02) and encompassed posterior cingulate cortex, lingual gyrus, hippocampus, and pPaHC (Figure 3 and Table 4).

**TABLE 4:** Composition of significant clusters in a seed-to-voxel analysis comparing aged users vs. aged non-users, using bilateral cerebellar lobules IV/V as seeds.

Cluster 1		
**Coordinates**	**Total voxels**	***p*-FWE**

(–14, –50, +10)	664	0.0039

**Number of voxels**	**Percent coverage**	**Region**

149	3	Precuneous
85	4	Posterior cingulate
84	6	Lingal gyrus, L
80	21	posterior parahippocampal gyrus, L
36	4	Cerebellum lobes IV/V, L
23	3	Hippocampus, L

**Cluster 2**		

**Coordinates**	**Total voxels**	***p*-FWE**

(+12, –44, +02)	444	0.038

**Number of voxels**	**Percent coverage**	**Region**

125	5	Posterior cingulate
99	6	Lingual gyrus, R
25	4	Vermis IV/V
19	6	posterior parahippocampal gyrus, R
17	2	Hippocampus, R
15	2	Cerebellum lobules IV/V, R
12	5	Vermis III
6	3	Cerebellum lobule III R

*Coordinates are (x, y, z). Regions with 1% coverage or greater are reported.*

## Discussion

In the present study, we describe differences in rsFC in older adult cannabis users relative to non-users. Notably, we find greater connectivity between the anterior cerebellum and two regions: hippocampus and pPaHC, with pPaHC cortex having stronger connections to lobule IV/V and vermis IV/V, and hippocampus having stronger connections to vermis IV/V and cerebellar lobule III. This strengthening of connections between hippocampal and parahippocampal regions and anterior cerebellum is also seen in 25–35-year old-non-users compared to older non-users.

The alterations in rsFC in the cerebellum with the hippocampus and with the parahippocampal cortex are consistent with anatomical studies that identified high levels of cannabinoid receptors in these regions (Herkenham et al., 1990). The endocannabinoid system is influenced by age. For example, CB1 receptor binding and gene expression decline with age in the cerebral cortex, limbic structures, and hippocampus in animal models (Berrendero et al., 1998) and humans (Di Marzo et al., 2015). Some researchers argue that the endocannabinoid system directly controls aspects of the aging process, such that CB receptors control the cellular processes of age-related inflammation, and that age-related declines in CB1 and endocannabinoid levels induce the declines in cognition common in old age (Di Marzo et al., 2015). These arguments are bolstered by animal studies showing that adult, but not juvenile, CB1-null mice perform worse than wild-type mice in a variety of cognitive studies. Further, age-dependent cognitive declines in CB1 mutants are accompanied by increased age-related hippocampal neuroinflammation and accelerated loss of hippocampal neurons (Bilkei-Gorzo et al., 2005; Albayram et al., 2011; Bilkei-Gorzo, 2012). A recent animal study by Bilkei-Gorzo found that low doses of THC administered to older mice increased hippocampal spine density as well as molecular markers associated with synaptogenesis (Bilkei-Gorzo et al., 2017). These anatomical changes in the hippocampus were accompanied by changes in cognition, such that cognitive performances attained in treated old mice were indistinguishable from those in untreated younger mice. Other studies have found improved spatial memory, decreased age-related inflammation, and increased hippocampal neurogenesis in old rats after administration of a CB1 receptor agonist (Marchalant et al., 2008, 2009). Though it is premature to assume that these findings can be directly translated to human brain development and function, these findings are consistent with our data showing that cannabinoid consumption is associated with age-related changes in brain connectivity, particularly in the hippocampus and cerebellum.

Both the cerebellum and hippocampus are highly sensitive to the effects of aging. For example, compared to cortical regions, which show gradual linear decline with advancing age throughout adulthood, hippocampal volumes are stable until around age 50, at which point the hippocampus undergoes a rapid period of volumetric decline (Fjell et al., 2013). This is especially important given the relationship between hippocampus and age-related cognitive impairment and Alzheimer’s disease (AD). Several studies have demonstrated that hippocampus and entorhinal cortex are smaller in individuals with AD and individuals with mild cognitive impairment relative to healthy controls (Bell-McGinty et al., 2005), with one study reporting volume reductions of 39% in the entorhinal cortex and 27% in the hippocampus (Du et al., 2001). Cerebellar volumes are also decreased in aging, particularly in the anterior regions (lobules I–V) of the cerebellum (Hulst et al., 2015). Moreover, these same cerebellar subregions are correlated with performance on sensorimotor and working memory tasks among older individuals (Bernard and Seidler, 2013). Reduced rsFC between hippocampus and cerebellum has also been found in Alzheimer’s patients (Allen et al., 2007). In the current study, these anterior cerebellar regions were found to have stronger hippocampal connections in older adult cannabis users as compared to non-users.

Our findings should be considered in the context of some limitations. First, this is a cross-sectional analysis of groups of individuals who either currently used cannabis or did not. Thus, we can make no *causal assertions* about whether cannabis use induced positive changes in connectivity, prevented the normal age-related decline in connectivity, or if there is a third variable at work here influencing the observed differences in connectivity between the groups. Second, we performed a coarse categorization of users versus non-users based on the available data, so we were unable to conduct any dose-response relationship regarding functional network connectivity and the duration, frequency, or amount of cannabis use. Finally, the older adults in our sample are a very healthy group who volunteered for a supervised exercise study. Exclusion criteria were rather stringent, and this was a largely White, educated, and high SES sample, so it is not clear whether our findings would generalize to the broader older adult population.

Collectively, these findings suggest the intriguing possibility that, consistent with the pre-clinical animal literature, there may be some benefit of cannabis use for the aging human brain. Future studies, particularly longitudinal studies that follow older adult cannabis users and non-users over time to examine *changes* in connectivity in concert with fine-grained data on quantity, frequency, and potency of cannabis use, will be critical to understand the potential harms and potential benefits of cannabis use for brain health and cognitive function among older adults.

## Data Availability Statement

The raw data supporting the conclusions of this article will be made available by the authors, without undue reservation.

## Ethics Statement

The Institutional Review Board of the University of Colorado Boulder approved all study procedures for both datasets (Protocol 13-0392 for dataset 1 and 15-0457 for dataset 2). The patients/participants provided their written informed consent to participate in this study.

## Author Contributions

AB, RT, and KH designed the experiment. RT gathered data. KW, AB, RT, CS, and KH generated hypotheses. KW performed analysis. All authors wrote the manuscript, contributed to the article, and approved the submitted version.

## Conflict of Interest

The authors declare that the research was conducted in the absence of any commercial or financial relationships that could be construed as a potential conflict of interest.

## Publisher’s Note

All claims expressed in this article are solely those of the authors and do not necessarily represent those of their affiliated organizations, or those of the publisher, the editors and the reviewers. Any product that may be evaluated in this article, or claim that may be made by its manufacturer, is not guaranteed or endorsed by the publisher.
