# Supplementary material for: Cannabis Use and Resting State Functional Connectivity in the Aging Brain
Source: Front Aging Neurosci. 2022 Feb 10;14:804890. doi: 10.3389/fnagi.2022.804890 (PMC8868145; doi:10.3389/fnagi.2022.804890)
Supplement: Supplementary file 1 [file Table_1.pdf]

Medication	Older Users (n=43)	Older Nonusers (n=153)	Younger Nonusers (n=23)
**Statin**	8 (18.6%)	33 (21.6%)	0
**Thyroid Medication**	5 (11.6%)	44 (28.8%)	0
**Proton Pump Inhibitor**	1 (2.3%)	8 (5.2%)	1 (4.3%)
**Antidepressant**	8 (18.6%)	32 (20.9%)	3 (13.0%)
**Angiotensin II receptor blocker**	3 (7.0%)	17 (11.1%)	0
**Calcium channel blocker**	1 (2.3%)	9 (5.9%)	0
**Beta blocker**	1 (2.3%)	8 (5.2%)	0
**Non-steroidal anti-inflammatory**	2 (4.7%)	9 (5.9%)	1 (4.3%)
**Angiotensin-converting enzyme inhibitor**	2 (4.7%)	20 (13.1%)	0
**Hormones**	2 (4.7%)	8 (5.2%)	2 (0.09%)
**Supplements**	1 (2.3%)	3 (2.0%)	0
**Other**	6 (14.0%)	53 (34.6%)	1 (4.3%)

Table S1. Medication use across subject groups. Antidepressants included SSRIs, SNRIs, and tricyclics. Hormones included estrogen, progesterone, and testosterone replacement therapy, as well as birth control pills. “Other” included anti-seizure medications, anticoagulants, diuretics, and other miscellaneous medications.
