# Supplementary material for: Cannabis Use and Resting State Functional Connectivity in the Aging Brain
Source: Front Aging Neurosci. 2022 Feb 10;14:804890. doi: 10.3389/fnagi.2022.804890 (PMC8868145; doi:10.3389/fnagi.2022.804890)
Supplement: Supplementary file 2 [file Image_1.pdf]

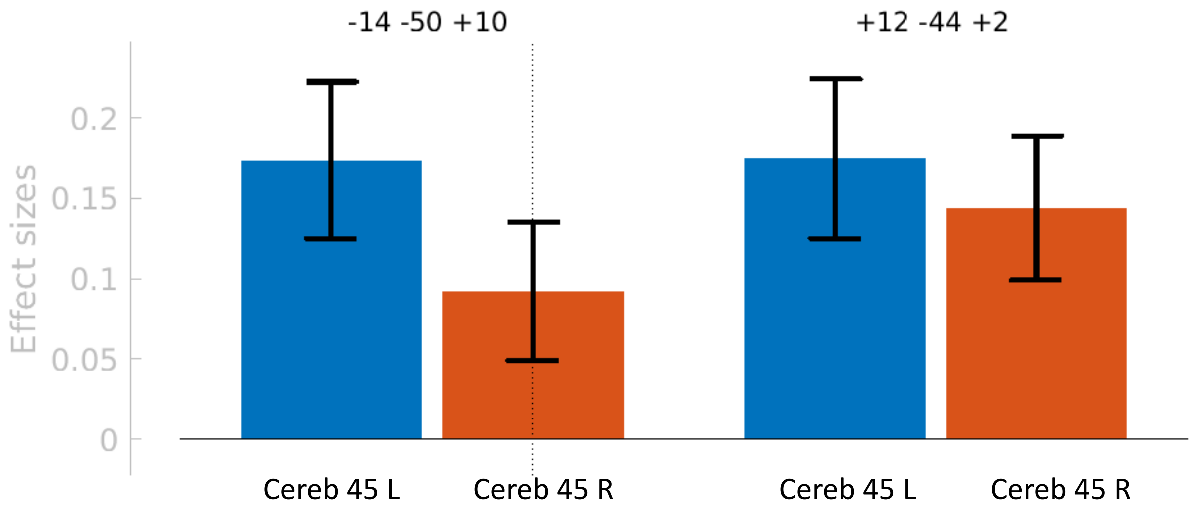

Figure S1. Effect sizes of differences between Older Users and Older Nonusers for seed-to-voxel analysis using cerebellar lobules IV/V (Cereb 45) as the seed. Positive values indicate stronger correlations between the seed and cluster for Older Users relative to Older Nonusers. Clusters are fairly symmetric and include bilateral posterior cingulate cortex, lingual gyrus, hippocampus, and pPaHC (Figure 3).
